# Pressure Algometry for the Detection of Mechanical Nociceptive Thresholds in Horses

**DOI:** 10.3390/ani10122195

**Published:** 2020-11-24

**Authors:** Kevin K. Haussler

**Affiliations:** Equine Orthopaedic Research Laboratory, Department of Clinical Sciences, College of Veterinary Medicine and Biomedical Sciences, Colorado State University, Fort Collins, CO 80523, USA; kevin.haussler@colostate.edu

**Keywords:** pressure algometry, mechanical nociceptive thresholds, nociception, pain, pain models, pain detection, repeatability, horse

## Abstract

**Simple Summary:**

It is difficult to measure pain in horses. As animals are not able to verbalize what they feel, we are left with trying to interpret the different signs that they display when they are in pain. Many of these signs are vague (e.g., not eating their food), but some are more readily identified if the animal moves away or lifts their leg when pressure is applied to a sensitive area. Pressure algometry is a tool used to detect responses to applied mechanical stimuli within painful and nonpainful tissues. Pressure algometry has been used in many different studies, but there is no consensus on how to synthesize this information to better diagnose and treat pain in horses. The purpose of this study was to summarize the results of these studies. Based on that review, we conclude that there is good evidence that pressure algometry is a reliable and objective method to measure pain responses. This information will help to improve the diagnosis and treatment of pain in horses.

**Abstract:**

The clinical assessment of pain is subjective; therefore, variations exist between practitioners in their ability to identify and localize pain. Due to differing interpretations of the signs or severity of pain equine practitioners may assign varying levels of clinical significance and treatment options. There is a critical need to develop better tools to qualify and quantify pain in horses. Palpation is the most common method to detect local tenderness or sensitivity. To quantify this applied pressure, pressure algometry has been used to gradually apply pressure over specified landmarks until an avoidance response is noted, which is defined as the mechanical nociceptive threshold (MNT). Numerous studies have used pressure algometry in different applications to measure MNTs in horses. There is an acute need to establish normative values within different body regions and to develop standardized methods of testing MNTs to better guide practitioners in the diagnosis and treatment of pain. The aim of this systematic review was to summarize the evidence for the use of pressure algometry in horses. There is good evidence that pressure algometry is a repeatable, semi-objective method that can be used in a wide array of clinical and research applications to assess MNTs in horses.

## 1. Introduction

**Figure 1:**
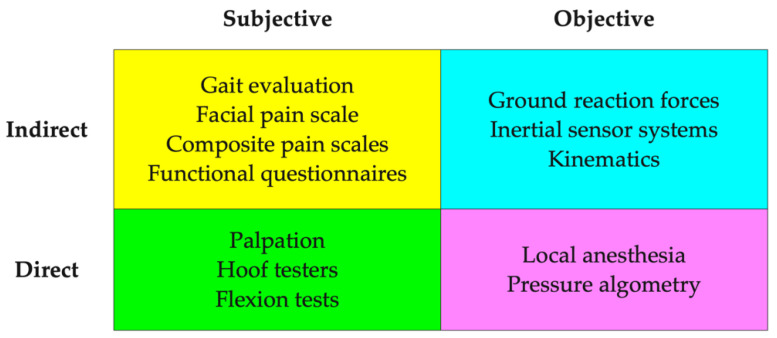
Categorization of methods used for pain detection.

The clinical assessment of pain in horses is always subjective. Large variations can exist between practitioners in their ability to identify and localize pain, as well as, offering differing interpretations of the assigned clinical significance [1,2]. In addition, serial assessment of musculoskeletal pain is highly variable even within the same patient or examiner [3]. There is a critical need to develop tools to better qualify and quantify pain in horses. General categories of pain assessment include subjective versus objective and direct and indirect (Figure 1). Subjective methods tend to rely more on qualitative measures of signs of pain, whereas, objective measures often are able to quantify the presence or absence of pain (e.g., diagnostic anesthesia) or assign a numerical number to altered gait or local pain thresholds. Indirect pain measures evaluate global or systemic signs of pain, whereas, direct measures often use applied pressure or local anesthetics to identify and localize tissue sensitivity. 

Pain models have been developed for use in horses as a method to quantify nociceptive thresholds (Table 1). Many of these models have been used in combination to determine their relative sensitivity and specificity. For musculoskeletal pain in horses, the best results have been reported, in descending order, for mechanical, thermal and electrical stimulation [4]. From a clinical perspective, soft tissue and bony palpation is the most common method to detect local tenderness, which is the primary manifestation of most, if not all musculoskeletal pain [5]. Palpation provides the most useful and relevant information about both the underlying tissues (e.g., heat, swelling) and the nociceptive system [6].

Pressure algometry is used to assess mechanical nociceptive thresholds (MNTs), which are defined as the minimum pressure that induces pain or a pain response [14]. Lower MNT values indicate more sensitivity and higher MNTs indicate less pain. Mechanical pain-sensing neurons include both nociceptive (C fibers) and mechanonociceptive (Aδ) nerve fibers that terminate as free nerve endings within the skin [15,16,17]. These receptors are heterogeneous and can modify their response to mechanical, thermal and chemical stimuli, depending on the local inflammatory milieu. Mechanoreceptors often become more responsive to nociceptive stimuli in the presence of heat and chemical mediators. Over the last decade, there have been numerous applications of using pressure algometry to measure MNTs in horses [18,19,20,21,22]. There is a critical need to establish normative values within different body regions and to develop standardized methods of testing MNTs. The aim of this systematic review is to summarize the evidence for the use of pressure algometry in detecting MNTs in horses.

## 2. Materials and Methods

Searches were conducted to identify all published articles on the use of pressure algometry to measure MNTs in horses. PubMed, Web of Science, and Google Scholar databases were searched from their respective inception until October 2020. The literature search was designed to retrieve all articles with keywords of horse, pressure algometry, and mechanical nociceptive threshold. Additional hand searches were conducted of conference proceedings and bibliographies of all retrieved articles. There were no restrictions on the language of publication.

All studies that included methodology, establishing normative values, use of pain states, response to diagnostic analgesia, and any measured response to therapy were included. Studies using foals or donkeys, dogs, cats, sheep and pigs were excluded. Studies that used a single point application to the dorsal metacarpal region to assess nociception in pharmacologic studies were excluded as they provided few additional insights into the application of pressure algometry in horses. Articles were read in full, and data were extracted systematically in a predefined, standardized manner according to design, technical aspects, application of the device, operator bias, subject variability, safety and avoidance reactions, normative values, and diagnostic and therapeutic applications.

## 3. Results

There were 26 studies that met the inclusion criteria (Table 2).

### 3.1. Application in Horses

Horses, as with all animals, are non-verbal and are not able to vocalize pain sensations or intensity. The most common nociceptive stimuli that are likely to negatively impact horses originate from the external environment in the form of mechanical stimuli such as lacerations, bites, kicks, or pressure from a rider or tack. As riders and trainers use applied pressure as training aids to model desired movements, most horse’s initial response to applied pressure is to move away from the initiating source. However, with additional training horses learn to interpret the applied pressures as signals to perform certain tasks instead of engaging in an instinctual flight response if the stimulus is applied too forcefully or suddenly [44]. These learned responses of standing still while non-noxious or low-intensity noxious stimuli are applied, form the basis for the use of pressure algometry to measure MNTs in horses. As nociceptive thresholds and the response to noxious stimuli are individually based, pressure algometry may be more useful for the quantification of intra-subject responses, rather than a method for assessing nociceptive differences between horses. As with any clinical measure, a collection of subject and objective assessments should be collected from a whole horse perspective and the sole reliance on a single tool or parameter to make diagnostic or therapeutic decisions is discouraged. Mechanical nociceptive thresholds should be combined with other clinical and available indicators of musculoskeletal pain, such as lameness, temperature changes of the skin, muscle hypertonicity, joint hypermobility or hypomobility, imaging techniques, and the use of diagnostic local anesthesia [25]. Pressure algometry has been used in numerous studies to evaluate its repeatability within and between examiners and over varying time intervals [24,25,27]. In general, intra-examiner repeatability is reported to be good and inter-examiner repeatability often depends on the level of operator experience with using the instrument [25]. The benefits of pressure algometry are that it is a relatively inexpensive, portable, repeatable, and semi-objective technique to assess nociceptive thresholds in horses.

### 3.2. Technical Considerations

As with any biomedical instrument, the technical aspects of a measurement device need to be fully understood to assure that it is safe to use and that it is actually measuring what it was designed for. Guidelines for proper application are also needed to ensure accurate and repeatable results. Any possible adverse effects or technical failures and how to limit or correct these issues need to be disclosed. The following content outlines the reported technical considerations for using pressure algometry to assess MNTs in horses.

#### 3.2.1. Pressure Algometer Features

##### Definitions—Force, Pressure, Surface Area, Units

Unfortunately, there no established standards for reporting MNT values due to differences in the methods used and the mixed reporting of pressure and force [15]. Force is defined as any interaction that, when unopposed, will change the motion of an object and can be described as having a magnitude and direction. Force is typically measured in Newtons (N) where 1 Newton is defined as the amount of force needed to accelerate 1 kg of mass at the rate of 1 m/s^2^. Pressure is defined as the amount of force applied perpendicular to the surface per unit area and can be measured in different units, but is typically reported in Pascal or 1 N/m^2^ [41]. As most methods in horses use a small surface area to apply a noxious stimulus then pressure is often reported as kg/cm^2^, which is more intuitive as a clinical measurement as 1 kg/cm^2^ = 98,066 Pascal (Pa) or 98 kPa. Due to the design of some studies and the methods used to induce MNTs, some authors chose to report values only as force without providing the known contact area that the force is applied. This limits the external validity of the study as the intensity of the stimulus (i.e., pressure) and therefore the results cannot be readily compared to other studies [15].

##### Methods Used to Apply Pressure

There are two general methods used to apply mechanical stimuli in horses, which include either a movable hand-held or a static limb-mounted device. Hand-held devices are easy to use and can be applied to any surface (i.e., appendicular or axial skeleton) and tissue type (i.e., over bone or muscle) [19,21]. Device handling is an important feature to consider when using hand-held devices as the size, shape and ability to grasp the device and to generate the consistent forces needed to produce reliable MNT measurements is critical. Limb-mounted devices can either use a pressure cuff or a pneumatically-driven pin to apply noxious stimuli and have the benefit of more controlled force application without the presence or cues associated with an operator in the immediate vicinity of the animal [15]. Some studies have used blindfolds in horses to limit environmental distractions and the visual perception of the applied device [22]. Limb-mounted devices seem to be the most preferred system for pharmacodynamic investigations of analgesic agents [4,45,46]. Hand-held and limb-mounted devices have been compared in pigs and the hand-held algometer resulted in lower MNTs, compared to a limb-mounted actuator with an identical probe tip in both normal and lame adult sows [47]. All methods suggest a perpendicular oriented application of force as this helps to prevent unwanted tissue slippage and risk of injury and also provides a more accurate measure of the applied pressure (i.e., force vector is oriented perpendicular to the surface).

##### Probe Tip Configurations

The probes used to induce noxious stimuli differ in type, size, shape, and number, which adds a source of variability and can makes comparisons across studies difficult [23]. Probe tips typically consist of small metal cylinders or have attached rubber tips to limit sharp edge transitions and reduce slippage, which it thought to improve comfort and reduce the risk of injury [15,24].

Different probe sizes are considered to produce varying effects due to differences in tissue penetration depth and types of superficial or deep nociceptive fibers stimulated [23,48]. Small tips (e.g., 1 mm diameter) have been shown to produce more consistent results, due to very focal pressures and the preferential stimulation of nerve endings in the skin or over bony landmarks [15,34]. The most common sizes of probes are 0.5–1 cm^2^, which are thought to induce a summation of nociceptive responses within the superficial skin and underlying muscle or fascial layers [23]. Probes sizes >1 cm^2^ often produce more variable results, due to the application of very diffuse, low pressures that require the application of much higher forces to stimulate a comparable nociceptive response [48,49]. The application of higher forces may also simply move the animal or the limb, thus preventing detection of true nociceptive thresholds [50].

Numerous probe shapes have been used, but most consist of a round cylinder with sharp edges or a hemispherical tip that has rounded edges [23]. The sharp edges of metal cylinders have an increased risk of localizing pressure and increasing discomfort, or the risk of tissue injury when used repetitively. Round-tipped probes have been reported to produce lower MNTs than flat-ended probes [15]. Other shapes such as cone tips have been used in the hoof region to test nociceptive thresholds where higher pressures might be indicated in highly keratinized tissues [22,36].

Most studies use a single probe tip; however, multiple probe configurations have also been used in an effort to maintain better skin contact and to prevent probe angulation and tissue slippage. However, there is the potential for one pin to make skin contact before the remaining two pins and thus the applied force can be inconsistent. Increased variability in MNTs values has been reported when using a three-pin actuator, compared to a single probe tip [15,51].

##### Analog Versus Digital Readouts or Controls

Devices with analog displays are typically less costly, easily held in the palm of the hand, and lend themselves to customizable configurations within hand-built systems. Digital readouts or and systems that use computer-driven actuators can provide more accurate results but are often more costly and cumbersome to use in field settings [15,52]. Digital devices may also provide the option for monitoring the rate of pressure application, which has been shown to be a critical aspect in measuring MNTs in horses [53,54].

##### Calibration

As the majority of studies use commercially available instrumentation, calibration of the units is provided by the manufacturer and is not a feature that can be readily adjusted by consumers on most devices [27]. Most devices used to measure MNTs are reported to be stable and do not need constant recalibration [54,55]. However, a few manufacturers do provide a standard weight [28,40] or recommend using a manometer [15] for calibration of the instrument prior to recording. While drift in measured MNT values could be expected, the clinical relevance is likely negligible as there are no accepted normative MNT values across body regions within horses [24]. The most clinically relevant aspect of pressure algometry is to measure changes within an individual patient and slight changes in calibration are not likely to alter the clinical interpretation of MNT values over short periods of time.

#### 3.2.2. Application of the Device

##### Environmental Considerations

MNTs should be recorded with minimal restraint in a quiet area in a routine and systematic manner to limit any external factors from influencing the horse’s attention or mental state. In an effort to keep horses calm during the MNT testing, several researchers have acclimated horses to the testing environment or have kept them in their own natural environment to avoid conditioned fear or pain responses [27]. To limit environmental distractions and the visual perception of the algometer being applied, a blindfold has been applied to horses during MNT testing [22]. As horses are socially bound to living in herds, some researchers test MNTs in the company of another restrained horse to avoid stress and anxiety related to social isolation, which have the potential to negatively influence MNTs [15]. Although, MNT measurements in donkeys are reported to not significantly differ in the presence or absence of a companion donkey [49].

##### Site Selection

Pressure algometry has been used to assess MNT across a wide number of anatomical landmarks in horses (Table 3). Within the axial skeleton, the trunk region has been most commonly used to assess MNTs at both bony and soft tissue landmarks. Within the appendicular skeleton, the metacarpal region has been used mostly to assess analgesic effects in pharmaceutical-related studies due to the ease of instrumentation with a limb-mounted device and the lack of overlying muscles or other soft tissues [34]. There are limited published reports comparing fore versus hind limb MNTs in horses. In one report, there were no significant differences between fore and hind limb MNTs [49]. However, when an electrical stimulus was used there were lower nociceptive thresholds measured in the forelimb, compared to the hind limb [56].

MNTs have been recorded in both unweighted and weighted limbs [30,33]. Most pharmaceutical studies test MNTs in weighted forelimbs as the horse can stand quietly restrained during the testing procedure, which may help to improve identification of subtle avoidance reactions (e.g., weight-shifting to the non-tested limb) [51]. The ability to stand on all four limbs during testing helps to reduce environmental and iatrogenic stresses associated with restraining the limb while attempting to measure MNTs. However, specific site testing within the distal limb may be safer and better accomplished in the unweighted limb. Theoretically, MNTs would be expected to be lower within unweighted limbs because of the ease by which the horse can produce an avoidance response (e.g., pull the limb away), compared to a weightbearing limb [19].

Site selection within the appendicular skeleton is important from both safety and repeatability perspectives. Most studies use the thoracic limb, versus the pelvic limb, due to ease of instrumentation and safety considerations. Site selection should also consider the different withdrawal responses of the fore and hind limbs. In the forelimb, the natural response is to lift the limb in a flexed posture. Therefore, testing MNTs along the dorsal aspect of the forelimb induces forces that might delay or limit forelimb flexion; whereas, testing sites along the lateral or palmer surfaces (e.g., flexor tendons) may actually precipitate reflexive forelimb flexion solely due to a learning response to applied pressure in this region and not due to a true nociceptive threshold [30]. Within the pelvic limb, the natural response to applied pressure for some horses is to instinctively kick out instead of gently withdrawing the limb. Pressure applied at sites on either the dorsal or plantar surfaces of the hind limb may produce similar unsafe responses as kicking out [32].

Hair coats in horses are typically short enough to not influence MNTs; however, some studies have clipped hair with the goal of reducing potential artifacts and to improve repeatability [15], although, mean MNTs are reported to not be significantly different when measured either with the hair intact or clipped [49]. Caution is needed as the simple act of clipping hair can cause skin irritation in some horses. For longer term studies that required repeated testing at the same site over time, marking or clipping small areas of the skin to demark testing sites has been useful [42].

##### Rate of Pressure Application

The rate of pressure application is deemed an important technical aspect of determining MNTs in horses as too slow of rates may only aggravate the horse and too fast of rate may override any noticeable signs of nociceptive thresholds [49]. Although, a slow or constant rate of pressure application is likely to produce more precise MNTs measurements [60]. The challenge with most algometry devices is that there is no consistent way to measure or monitor the rate of pressure application unless mechanical actuators under computer control are incorporated [15]. It is difficult to manually apply a constant rate of pressure without extensive training and experience (i.e., learning curve) [25]. Unfortunately, no standard rates of pressure application have been established for horses. In humans, rates typically vary from 0.2 to 1 kg/cm^2^/s [61]. In horses, the reported range of pressure application typically vary from 3 to 10 kg/cm^2^/s; however, rates of 5-10 kg/cm^2^/s seem to produce the most repeatable results without masking withdrawal or avoidance responses [24,25,41]. As many MNT values can be quite high within the axial skeleton (30–40 kg/cm^2^), applying pressure at a slow rate of 2 kg/cm^2^/s would require pressure application over 15–20 s per test, which is not feasible from a study perspective and not tolerated by most horses. However, when testing painful sites, then much lower MNTs are expected and a slow rate of 2 kg/cm^2^/s may be ideal [20,29].

##### Recording MNT Values

Single tests without replication are performed in most pharmaceutical studies assessing MNTs within the distal forelimb using a pressure cuff and indentation probe [62,63]. The most common approach for MNT testing within the axial skeleton is to calculate the mean of three replicates taken at 3–4 s intervals [31,42]. If one value differs substantially from the other two (i.e., exceeds the normal variability) due to sudden movement by the horse or skin slippage with the probe, then a fourth measure is typically recorded and the aberrant value discarded [18,57].

In most studies, a fixed-order protocol is used to test multiple, bilateral sites in an effort to reduce between-subject variability (i.e., the same sites are measured in the same order for all horses) [24,27]. There has been a reported significant effect of the sequence of testing; however, this was judged to be most related to increased variability in measured MNT values [18].

Most studies use a single examiner to measure MNTs and the readings are transcribed by a second observer [19,24]. It is important that the examiner does not view the MNT values during testing as it is easy to be influenced by focusing on the applied peak force and distracted from accurately visualizing any avoidance responses [39,41]. In an effort to reduce additional bias, some studies have one examiner measure MNTs and then hand the instrument off to an observer for recording so that the examiner is blinded to the recordings [25,26,42]. As a further precaution, two additional observers have been used to evaluate the horse’s response to the applied pressure to judge if the proper nociceptive endpoint was attained by the examiner during the testing procedure [22].

##### Instrument Repeatability

When using any instrument to measure biological parameters, there are three general categories associated with variability: the instrument itself, the examiner, and the subject. Measures of instrument repeatability across axial skeleton sites report a median range for three consecutive measures of 1–2 kg/cm^2^ [24,32,39]. Mean MNT values have also been shown to have remained stable over a 3-week period [49].

#### 3.2.3. Technical Failures

Most studies implement an upper limit or threshold to pressure application in an effort to prevent potential tissue damage [22,34]. However, in other studies, the maximum limit (i.e., ceiling effect) of the instrument is reached prior to a nociceptive response is noted, thus preventing collection of a complete data set [19,35]. It is important for investigators to do preliminary investigations into the lower and upper limits of the instrument and the specific tissues to be tested to optimize the accuracy the MNT testing. Ideally, collected MNT values should be normally distributed within the midrange of the instruments testing capabilities.

Stabilization of the probe tip is often required in both axial and appendicular applications to acquire accurate MNT readings as slipping of the probe tip often induces a local tissue response due to unwanted tissue shear versus tissue compression. While skin bruising has been reported in humans with repeated measurements at the same site [64], no signs of local inflammation or bruising have been reported in horses despite attaining much higher MNTs than recorded in human studies [24,49]. Full probe tip contact without tissue slippage is sometime difficult within the distal limb, especially over curved boney landmarks, such as the dorsal metacarpus [15]. Within the trunk region, the probe is often held between thumb and forefinger of the opposite hand to prevent accidental skin slippage [24,27]. For MNT testing within the cervical region, there are reports of the examiner stabilizing the contralateral side of the neck with the opposite hand or with an assistant providing physical support from the opposite side of the neck to reduce to effect of pushing the neck passively away with the applied force versus assessing a true nociceptive response [25,27].

### 3.3. Operator Issues

#### 3.3.1. Learning Curve

There is a documented learning curve or need to become familiar with the instrumentation and MNT testing procedure [19,27,31]. The primary issues that affect accuracy and repeatability are inconsistent rates of pressure application and poorly identified nociceptive responses or endpoints (i.e., stopping too soon or applying too much pressure). As there are no reported standards, the rate of pressure application is often based on a clinical impression of the horses’ response and the logistic limitations of the instrumentation or testing of multiple sites within a reasonable period of time across many horses [24]. Digital readouts of the rate of pressure application or metronomes have been used to improve consistency [25]. It is important to recognize avoidance reactions as in some settings these signs can be very subtle. Some studies have had examiners practice together to learn to better recognize avoidance reactions [25]. The application of pressure also needs to be stopped at the first sign of an avoidance reaction to accurately capture nociceptive thresholds [26,27,65]. As an additional precaution, some horses may be overly sensitive and may react violently to the applied pressure, thus creating unsafe and undesired responses, especially if repeated measures are required [27].

#### 3.3.2. Hand Dominance

It is reasonable to expect that hand dominance and dexterity (i.e., ability to firmly grasp the instrument) could play a role in the ability to consistently apply peak forces if both left and right hands are used in MNT testing [66]. However, when tested across several examiners, no evidence of hand dominance has been reported in horses despite the application of a near maximal physical effort [19].

#### 3.3.3. Bias

Some level of bias is always present when using pressure algometry as the endpoint or threshold is determined by the subjective assessment of the examiner. In an effort to reduce bias, a supervised training period is recommended for the operator to become familiar with the equipment and the typical avoidance reactions exhibited by subjects within the test setting [25]. Additional precautions include applying pressure at constant rates and in triplicate at readily identified anatomical landmarks. Having the examiner blinded to the recorded MNT values is important during application of the pressure, so that all attention is focused on detecting the first sign of an avoidance reaction within the horse rather than observing changes in the displayed MNT values [24].

It is difficult to blind examiners when using pressure algometry at known pain or surgical sites; therefore, adjacent control sites are needed to provide an overall assessment of the individual subject’s nociceptive status (i.e., peripheral and central sensitization) and to determine the gradation of sensitivity around the lesion [31]. Slower rates of pressure application may be used unconsciously by examiners at known pain sites in an effort to limit the precipitating additional pain and tissue trauma. Avoidance reactions may also occur very rapidly and surprise the examiner when pressure is applied over inflamed tissues or in anxious horses that are hyper-reactive and anticipate the applied noxious stimuli. By including MNT sites that radiate away from the known pain or surgical sites, a more objective assessment of the clinical relevance of the measured MNT values can be determined even in unblinded observers [29,67].

#### 3.3.4. Intra- and Inter-Examiner Repeatability

Intra-examiner repeatability depends on the body region tested and the presence or absence of pain (Table 4) [31]. Repeatability is also dependent on the examiner’s experience and on the duration (e.g., days versus weeks) between testing sessions. Intra-examiner repeatability varies from 2–3 kg/cm^2^ with a good interclass correlation (ICC) coefficient of 0.46 to 0.78 when measured at a 2-week interval [25].

Inter-examiner repeatability in normal horses has been reported as good (±2 kg/cm^2^) with an ICC coefficient of 0.64 [25]. In a pain setting, inter-examiner repeatability tends to be poor (ICC = 0.26) due to large fluctuations in nociceptive responses [20,29,31]. However, there is general agreement in that horses with high MNTs and horses with low MNTs are noted by most examiners, even if one examiner consistently records lower MNT values [25].

### 3.4. Subject Variability

#### 3.4.1. Signalment

Varying nociceptive thresholds within and between horses are thought be to related to genetic coding of neurotransmitters and neuromodulators that contribute to low and high pain thresholds and individualized responses to nociceptive stimulation (i.e., stoic versus exaggerated responses) [23]. Within the axial skeleton, higher MNT values have been reported in younger horses (<13 years of age; 59%), geldings (85%), heavier horses (>470 kg; 67%)), and ridden horses (100%) [24]. Within the thoracic limb, higher MNT values have been reported in geldings (100%), lighter horses (<390 kg; 80%) and taller horses (>146 cm wither height; 100%) [30]. Many different breeds and athletic disciplines of horses have been used in ridden and unridden settings to measure MNTs, but no clear differences have been reported due to the use of inconsistent testing methods across studies.

#### 3.4.2. Exercise Effects

There are consistent and substantial increases in MNTs reported for actively ridden horses, compared to unridden horses [24]. In horses used within a collegiate program, MNTs measured along the dorsum of the trunk were lowest at the beginning of the semester and highest after 2 months of training and active competition [68]. MNT values then decreased 1 month after cessation of active competition while horses continued in routine activities. Within actively ridden horses, there are no reported significant effects of the timing of the exercise relative to the recorded MNT values [39]. However, as a precaution, some researchers have scheduled MNT testing either before or at least 4 h after routine exercise [18].

#### 3.4.3. Diurnal Fluctuations

The time of day appears to influence pain perception [23]. Diurnal variations have been reported in MNT values where the morning values (7.4 kg/cm^2^) versus evening (6.9 kg/cm^2^) MNT values were significantly different [27]. As a proposed mechanism for diurnal variations in nociception in horses, a positive correlation has been reported between cyclic changes in beta-endorphin levels and thermal nociceptive thresholds [69].

#### 3.4.4. Acclimatization

There is a slight acclimatization period to acquiring MNT measurements in horses as most instinctively step away from applied pressure [24,37]. The assessment and scoring of the overall mental status (e.g., very calm to extremely nervous) and tolerance to the procedure (e.g., readily tolerated to unable to test) have been used to measure horse responses to MNT testing [19]. Horses with high initial mental status scores have been reported to have lower mental scores after repeat MNT examinations. Calm or stoic horses could be expected have higher MNTs compared to anxious horses, but this has not been confirmed. In some nervous or fearful horses, there may be an initial startle or reflex that is not associated with a nociceptive threshold [34]. In an effort to improve repeatability, some studies have used an acclimation period to help horses become accustomed to the equipment and its use [15]. Other researchers have used adjacent control sites to familiarize horses to the pressure testing procedures [18]. Once horses have become accustomed to the MNT technique, they often stand quietly with predictable and uniform endpoints readily identified. However, in some painful horses the anxiety of pressure testing in a region of increased sensitivity may prevent the application of pressure algometry (Table 5) [20,28,30].

#### 3.4.5. Response to Repeated Measures

Sensitization refers to a horse becoming overly sensitive to a repeated stimulation; whereas, habituation occurs when a there is a muted or absent response to repeated stimulation. Both of these concepts have been tested extensively in MNT studies as they can impact study results. Sensitization could be expected in horses that anticipate or learn to avoid pressure by developing a premature response to noxious stimuli [23]. In studies that have reported sensitization (i.e., sequential decreasing MNTs) and habituation (i.e., sequential increasing MNTs) to three consecutive measures, there is a reported overall prevalence of 14% habituation, 15% sensitization and no consistent change or pattern in 71% of repeated measures (Table 6). There appears to be no clear pattern of habituation or sensitization across studies of normal or pain conditions.

An additional factor that can contribute to a consistent increase or decrease in repeated MNT measures, which can be perceived as patient sensitization or habituation, is the consistency by which the force is applied by the examiner and the ability to repeatedly identify a uniform endpoint. If the examiner is tentative and stops applying pressure before a clear endpoint is visualized with the first measurement, and then increases the pressure on subsequent consecutive measurements until a true nociceptive threshold is reached, this will be perceived as false patient habituation [37]. On the other hand, if the examiner is initially too forceful or does not realize that the patient is truly painful with the first measure, then less pressure is likely going to be applied during subsequent consecutive measurements and this would be perceived as false sensitization of the patient.

#### 3.4.6. Subject Repeatability

Within subject, left-right differences in MNT values have been reported to vary from <1 kg/cm^2^ to 3–6 kg/cm^2^, but are not shown to be significantly different at most sites within studies [27,32,39]. Overall within-horse variability at axial skeleton sites is reported to be ±1 kg/cm^2^ [24,25]. Due to differences in nociceptor concentrations within tissues and subsequent pain responses [65,70], it is expected that between-horse variability is higher and has been reported to be approximately 6 kg/cm^2^ [24].

### 3.5. Avodiance Reactions

Avoidance reactions or signs of pain noticed in response to MNT testing can be categorized into local, regional and systemic signs (Table 7). Many of the local reactions are body region dependent (e.g., trunk extension); whereas, the systemic signs are more generalized (e.g., stepping away) and are noted to be similar across studies.

### 3.6. Baseline Values

#### 3.6.1. Landmark Selection

The choice of sites to measures MNTs is an important consideration from both research and clinical perspectives. Some studies only test soft tissue (e.g., abdominal wall) [31] or epaxial musculature [26]; whereas, other studies typically measure MNTs over the metacarpal bone [71]. If the purpose of the study is to measure a treatment response, then it is important to also include distant control sites to better quantify any local treatment effects and differentiate them from other environmental or systemic influences on the recorded MNT values [29].

#### 3.6.2. Sites Differences

As pressure is applied over bony or soft tissue landmarks and tissues are compressed, skin sensitivity and the underlying tissue thickness are important factors in the production of MNTs [72]. The density of nociceptive receptors within different tissues contributes to differences in MNT values [6]. In vitro pressure testing in the skin and subcutaneous tissue samples show direct, nearly unattenuated pressure transmission [23]. Tissue thickness is positively correlated with pressure pain thresholds but also increases MNT variability due to diffusion of the applied pressures within the deeper tissues. Significant differences in spinal MNT values have been reported for landmarks over muscle, bone, and the dorsal midline of the trunk (Table 8). There are known MNT differences when measured by different examiners due to inter-examiner variability; however, general trends of MNT values within different tissues are noted.

#### 3.6.3. Spinal Region MNTs

Baseline values within regions of the axial skeleton have been reported to be lowest in the cervical region and highest in the pelvic region (Table 9). In the presence of known pain, local and regional MNT values are substantially reduced [29].

#### 3.6.4. Thoracic Limb MNTs

MNT values within the thoracic limb tend to be lower in the proximal landmarks and higher within the distal limb (Table 10). The type and diameter of the probe tip can have a significant influence on measured MNT values, which makes comparisons across studies difficult.

#### 3.6.5. Equine Digit MNTs

Pressure algometry has also been used to quantify MNTs within the equine digit (Table 11). Again, differences in probe tip sizes and shapes and reported MNT units make comparisons across studies difficult.

### 3.7. Diagnostic Applications—Pain Detection

#### 3.7.1. Local Anesthesia

As a method of evaluating the usefulness of pressure algometry for assessing nociception and pain processing, MNT responses have been evaluated in horses pre- and post-local anesthetic injections [22,35,36]. Following analgesia of the digital flexor tendon sheath in a single fore and hind limb, the mean MNT values for partially and fully desensitized limbs were reported to be significantly increased, compared with the noninjected contralateral limb (Table 12) [35]. There was a reported 2.9- to 5.6-fold difference in MNT values measured within the injected and noninjected limbs.

Following perineural injection of the palmer digital nerves, the mean MNT values within the region of desensitization increased significantly (Table 13). Using the same study design, intra-articular anesthesia of the distal interphalangeal joint did not produce any significant MNT differences at the same measured sites [22]. As there are known differences in local anesthetic diffusion [73,74], pressure algometry provides a useful tool to assess specific regions of desensitization.

#### 3.7.2. Diagnostic Nociceptive Thresholds

In humans, MNT values <3 kg/cm^2^ are often indicative of underlying pathology [5]. In horses with documented musculoskeletal disease, MNT values <5 kg/cm^2^ been deemed clinically significant [24]. In human subjects with lateralized pain, MNT differences >2 kg/cm^2^ are considered clinically significant with the painful side of the body having lower MNT values [5,14]. Similarly, left-right or painful-non-painful site comparisons with MNT differences >2.0 kg/cm^2^ are deemed clinically significant in horses [30].

#### 3.7.3. Nociceptive Repeatability

The repeatability of measured MNT values has been reported to be good for 3 consecutive measurements, left-right comparisons, and intra-examiner assessments, with more variability noted for inter-examiner repeatability [31]. Similarly, within-horse variability is reported to be less than between-horse variability [24,25]. Local anesthetics typically produce less MNT variability (e.g., CV < 0.20), compared to baseline values (e.g., CV > 0.50; Table 12) [35,36]. Conversely, painful conditions are often associated with increased MNT variability [28]. In horses with sacroiliac region pain the mean left-right differences were 8.1 ± 8.2 N/cm^2^ (CV = 101%), compared to a comparable control group with MNTs of 5.1 ± 3.4 N/cm^2^ (CV = 66%) [18]. In this same study, painful horses had pooled MNT values of 48.9 ± 13.9 N/cm^2^ (CV = 28%), compared to control horses with MNT values of 58.2 ± 8.9 N/cm^2^ (CV = 15%).

The reported mean differences and increase in variability in MNT values in painful versus normal conditions is due to alterations in nociceptive signaling and pain processing (i.e., sensitization) within individual subjects [23]. Nociceptive signals are processed via several peripheral mechanisms, which include transduction, transmission, and modulation within the spinal cord [75]. Transduction involves the conversion of noxious mechanical stimuli to electrical action potentials within the C and Aδ fibers via opening of mechanosensitive channels [17]. Persistent pain is often associated with physical or chemical changes that cause spontaneous firing and altered nerve conduction. Peripheral sensitization is characterized by the accumulation of inflammatory mediators that amplify nociceptive mechanisms. All of these processes are unique to the injury and to the individual; therefore, wide variations in MNT are expected across different tissue injuries and pain conditions in horses.

#### 3.7.4. Local and Regional Nociception

Pressure algometry has been shown to be useful in the identification and localization of naturally occurring or induced pain in a variety of clinical applications (Table 14).

MNTs measured along the ventral abdominal wall after midline celiotomy were 9.3 N/cm^2^, compared to baseline values of 12.0 N/cm^2^, which supports the clinical use of pressure algometry to detect pain in horses [31]. Differences in MNT values after intramuscular injections of sodium and procaine benzylpenicillin demonstrate the utility of pressure algometry in assessing tissue sensitivity (Table 15) [28].

Sensitivity due to microchip injection compared to sham (needle only) and control sites have also been measured with pressure algometry (Table 16) [20]. Small, statistically significant changes in MNT values could be measured, but the clinical relevance of these changes is unknown.

MNT values for experimentally induced pain at the T17-T18 and L3-L4 spinous processes were reported before and after Steinman pin placement [29]. A second session of pin placement at the same sites reflected a significant reduction in MNTs compared to the initial pin placement, indicative of local sensitization (Table 17).

Pressure algometry has also been used to track MNTs changes within the thoracic limb after an experimentally induced carpal fragment with subsequent osteoarthritis development over a 9-week period of time (Table 18) [30]. The percent change of mean MNT values across weeks in the osteoarthritic limb was −4%, compared to 7% within the contralateral control limb, which supports the use of pressure algometry to monitor long-term changes in MNT values.

#### 3.7.5. Systemic Nociception

Pressure algometry provides a method to semi-quantitatively measure responses to local limb perfusion [57,71] and various systemic medications [62,63]. The most common approach is to use a pressure cuff with an instrumented probe placed over the metacarpal region to induce single point pressure to detect nociceptive thresholds. As many of these studies use a small metal pin and only report force values in kg or Newtons, it is difficult to compare the results to other studies that report pressure values (i.e., force per unit area) [58].

### 3.8. Diagnostic Applications—Monitoring Treatment Effects

Pressure algometry has been used to assess the efficacy of different treatment modalities, with the majority focused on back pain (Table 19).

MNTs have been reported to be significantly increased (i.e., reduced pain) in horses after chiropractic treatment, compared to controls [37,39]. Within the treatment area 10 of 10 (100%) of landmarks had increased MNT values, compared to untreated regions of the axial skeleton where 11 of 19 (58%) of sites had increased MNTs. These findings support the idea that chiropractic treatment within the axial skeleton has both local and systemic effects. In a study comparing MNT values within chiropractic treatment, phenylbutazone, massage therapy, and active and inactive control groups, median MNTs 1-week post-treatment increased 27% within the chiropractic treatment group [39]. Smaller increases were noted within the massage therapy (12%) and phenylbutazone (8%) groups. Finally, the effects of chiropractic alone or combined with low-level laser therapy was compared with low-level laser therapy alone in horses with acute back pain [38]. In this setting, chiropractic treatment by itself did not produce any significant changes in back pain, whereas the laser therapy and combined laser and chiropractic groups had significant MNT increases after a series of three treatment sessions applied over one week.

Additional treatments for back pain that have been assessed with pressure algometry include tail traction, static magnetic blankets and extracorporeal shockwave therapy. In horses with clinical signs of back pain, tail traction produced significant increases in MNT values measured in the thoracic (83%), lumbar (50%) and pelvic (52%) regions, compared to baseline values [21]. In a blinded, placebo-controlled crossover study evaluating the effects of static magnetic therapy as applied in a blanket in horses without back pain, there were no significant differences noted between the active and sham treatment groups [40]. A double-blind, placebo-controlled, crossover study assessing the treatment of back pain with pulsed electromagnetic therapy applied within a blanket that consisted of two 10-day treatment session reported that active treatment only produced significant changes in MNT values at 5 of 25 (20%) of sites, which were not significantly different from the sham treatment group (6 of 25 (24%) sites) [41]. In horses with back pain treated with extracorporeal shockwave therapy three times at 2-week intervals, there were significant increases in MNT values in the thoracic region (64%) and lumbar region (29%) measured at day 56, compared to baseline [42].

The effect of ice immersion on the distal limb was assessed with pressure algometry applied to the pastern region with a 1-mm diameter probe and pneumatic actuator [34]. Surprisingly, there was no significant difference measured after icing (median MNT = 1.4 N (IQR 0.7–2.5)), compared to the baseline (median MNT = 1.6 N (IQR 0.7–3.2)), which might reflect that the skin, but not the deeper tissues were desensitized.

### 3.9. Regulatory and Equine Welfare Applications

Pressure algometry has also been shown to be of value in regulatory venues. MNT values were assessed within the distal limb of normal Tennessee Walking horses to establish normative MNT values to help with the regulation and prevention of soring (i.e., applied topical irritants or mechanical devices to induce exaggerated forelimb movements) [19]. The practice was banned by the Horse Protection Act in 1970 because of concerns about nonhumane treatment and responsibility for its enforcement is assigned to veterinary medical officers within the Animal and Plant Health Inspection Service (APHIS) of the US Department of Agriculture (USDA). Digital palpation of the distal forelimbs focuses on identifying the presence of pain by applying with enough pressure to partially blanch the thumbnail, which corresponds to approximately 2.3 kg of force (0.4–0.6 kg/cm^2^) [19]. MNTs were evoked by a pressure algometer at 4 sites within the pastern region by 6 different examiners. For the 5 veterinary medical officers, MNTs >10 kg/cm^2^ were reported in an average of 80% of the limbs, which suggests that the proposed 0.4–0.6 kg/cm^2^ of applied pressure is well below normative nociceptive thresholds and likely needs to be adjusted accordingly. Pressure algometry, in lieu of digital pressure, was judged to better quantify mechanical pressure applied during inspections to detect irritant therapy and improve consistency between examiners.

MNT testing applied during endurance racing has been reported to detect induced limb desensitization during competition [34]. The International Equestrian Federation (FEI) governs international sport and has specific regulations against modifying limb sensitivity (hyposensitive or hypersensitive) as they are considered performance enhancement procedures, which can lead to increased risk of injury or unfair competitive advantages. A study reported that 10 min after perineural local anesthesia (i.e., low-4-point block within the forelimb using mepivacaine) none of the horses responded to nociceptive stimulation up to the safety cut-off of 25 N, which supports MNT testing as a robust method for detecting limb desensitization [34]. Additional regulatory and equine welfare settings in which pressure algometry could be applied include hypersensitization within the metacarpal region of horses used in show jumping and hyposensitization of the sacrocaudal region to prevent unwanted tail movements in Quarter Horses [64].

## 4. Discussion

On the basis of this systematic review, evidence for the use of pressure algometry to detect MNTs in horses is compelling. As long as the technical considerations of applying the different instruments are understood, then the repeatability is consistent regardless of time, environment, or examiner. There are known operator bias and variations in the rate of application, but sufficient training and the use of a single examiner and independent observer to read the MNT values help to minimize these factors [43]. Knowledge of the expected range of MNT values for a specific application is important to select gauges that are able to capture the needed data without the risk of not detecting subtle nociceptive changes or exceeding the ceiling or maximum limits of the algometer. Sedation or a depressed mental state would obviously blunt or prevent avoidance reactions associated with detecting MNTs. Pressure algometry is able to detect areas of induced pain and areas of hypoalgesia, which lends credence to its clinical usefulness [33]. Due to the subjective nature of pain, poor definitions of acute and chronic pain in horses, and our limitations in defining a gold standard comparison, it is difficult to determine how sensitive pressure algometry might be in detecting acute versus chronic pain. Future research and the collection of MNT values from subjects with known pain will help to better define normative versus hyperalgesia parameters and improve the diagnosis and treatment of pain conditions in equine practice.

## 5. Conclusions

The current body of evidence suggests that pressure algometry is a repeatable, semi-objective method that is readily applicable in a diverse array of clinical and research applications to assess MNTs in horses.

## Figures and Tables

**Table 1:** Equine pain models and methods to assess nociception.

Stimulus	Application
Chemical	Synovitis [7]
Thermal	Radiant heat [8]
Electrical	Tooth pulp [9]
	Transcutaneous [10]
Mechanical	Hoof compression [11]
	Visceral dilation [12]
	Pressure algometry [13]

**Table 2:** Studies that used pressure algometry to assess mechanical nociceptive thresholds (MNTs) in horses.

Category	Application
Methodology	Probe tip configurations [15]
	Transmission of pressure through tissues [23]
Normative values	Normative values within axial skeleton [24]
	Normative values within distal forelimb [19]
	Intra-examiner and inter-examiner repeatability [25]
	Intra-examiner and inter-examiner repeatability [26]
	Inter-examiner repeatability [27]
Pain states	Intramuscular injections [28]
	Microchip insertion [20]
	Induced back pain [29]
	Induced carpal osteoarthritis [30]
	Celiotomy [31]
Diagnostic analgesia	Epidural morphine [32]
	Epidural ropivacaine [33]
	Low-4-point block [34]
	Digital flexor tendon sheath [35]
	Distal interphalangeal joint [22]
	Palmar digital nerves [36]
Response to therapy	Chiropractic [37]
	Chiropractic, low-level laser therapy [38]
	Chiropractic, massage, phenylbutazone [39]
	Static magnetic blanket [40]
	Pulsed electromagnetic therapy blanket [41]
	Extracorporeal shockwave therapy [42]
	Tail traction [21]
	Sublingual detomidine [43]

**Table 3:** Reported anatomical regions assessed with pressure algometry.

Axial Skeleton Landmarks	Appendicular Skeleton Landmarks
Entire axial skeleton [24,25,27]	Thoracic limb [30]
Neck [20]	Distal forelimb [15,22,36,45,51,57,58]
Trunk [23,26,29,33,38,39,40,41,42,43]	Pastern region forelimb [19,34]
Pelvis [18,21,33]	Pelvic limb [59]
Abdomen [31]	Tarsal region [32]

**Table 4:** Intra-examiner repeatability ranked across different body regions.

Site	Status	Mean Range
Neck, trunk and pelvis [24]	Normal	1.0 kg/cm^2^ (range 0–4)
Trunk and pelvis [33]	Normal	1.2 kg/cm^2^ (range 0–9.2)
Neck, trunk and pelvis [27]	Normal	1.3 kg/cm^2^ (range 0–4.8)
Trunk [37]	Ridden	1.4 kg/cm^2^
Trunk [39]	Ridden	1.6 kg/cm^2^ (range 0.7–2.9)
Neck, trunk and pelvis [25]	Normal	1.8 ± 1.4 kg/cm^2^ (CV = 78%)
Thoracic limb [30]	Carpal arthroscopy	1.8 ± 1.3 kg/cm^2^ (CV = 72%)
Thoracic limb [30]	Normal	2.0 ± 1.4 kg/cm^2^ (CV = 70%)
Pelvic limb [32]	Acute tarsal synovitis	2.2 ± 1.4 kg/cm^2^ (CV = 64%)
Distal forelimb—experienced [19]	Normal	2.1 ± 1.6 kg/cm^2^ (CV = 76%)
Trunk and pelvis [43]	Sublingual detomidine	2.6 ± 1.7 kg (CV = 65%)
Trunk [26]	Normal	4.1 kg/cm^2^
Ventral abdomen [31]	Celiotomy	6.6 ± 7.0 N cm^2^ (CV = 106%)
Trunk, pelvis [41]	Back pain	7.7 kg/cm^2^ (range 5.4–13.0)
Distal forelimb—inexperienced [19]	Normal	9.5 ± 0.3 kg/cm^2^ (CV = 3%)

**Table 5:** Ranked tolerance to applied pressure during MNT procedure.

Application	Prevalence	Intolerance
Neck and trunk—normal [27]	0 of 6 (0%)	na
Neck and trunk—normal [24]	0 of 36 (0%)	na
Distal forelimbs—normal [19]	1 of 26 (4%)	Inability to stand still
Distal forelimbs—normal [34]	0 of 108 (0%)	na
Ventral midline celiotomy [31]	1 of 23 (4%)	Kicked out—deemed unsafe
Microchip insertion [20]	1 of 18 (6%)	Hostile to all handling procedures Hypersensitivity to pressure
Intramuscular neck injections [28]	1 of 8 (12%)	Anxiety (repeated injections)
Thoracic limbs—carpal osteoarthritis [30]	4 of 24 (17%)	Refused to stand (carpal pain)

na = Not available.

**Table 6:** Proportions of habituation, sensitization, and no consistent pattern of change.^1.^

Sites	Habituation	No Pattern	Sensitization
Normal axial skeleton [24]	24%	68%	8%
Normal neck and trunk [25]			
Day 1, Examiner 1	20%	62%	18%
Day 2, Examiner 1	14%	70%	16%
Day 1, Examiner 2	10%	67%	23%
Day 2, Examiner 2	19%	66%	16%
Trunk (epidural) [33]	9%	72%	20%
Back pain [39]	20%	67%	13%
Back pain [37]	6%	68%	26%
Ventral midline celiotomy [31]	3%	83%	13%
Normal thoracic limb [30]	20%	72%	8%
Normal distal forelimb [19]	13%	70%	17%
Carpal arthroscopy [30]	16%	76%	8%
Acute tarsal synovitis [32]	17%	76%	8%
Average	14 ± 6%	71 ± 5%	15 ± 6%

^1^ Calculated across three consecutive MNT measurements.

**Table 7:** Avoidance reactions to pressure algometry applied across body regions.

Body Region	Avoidance Reactions		
	Locally	Regionally	Systemically
Head and neck [20,27]	Head shaking	Dropping the head	Stepping away
	Muscle fasciculations	Neck movement	Kicking out
	Biting		
Neck [28]	Neck stretching	Jaw movement	Kicking out
	Shaking the neck		Snorting
			Rearing
Dorsal trunk	Skin twitching	Trunk extension	Stepping away
[18,21,23,24,25,42]	Muscular contractions	Raised head	Looking at examiner
		Turn or flatten ears	Sudden lifting of limb
		Facial grimace	Stamping the foot
		Tail swishing	Kicking out
Ventral abdomen [31]	Skin twitch	Tail movement	Stepping away
	Increased muscle tone		Kicking out
Thoracic limb	Skin twitching	Muscle contraction	Stepping away
[15,19,30,34]	Muscle fasciculations	Weight-shifting to non-tested limb	Nosed at instrumented limb
	Lifting the limb		
	Pulls the limb away		
	Stomp foot		

**Table 8:** Mean spinal MNTs at different tissue locations.

Condition	Bony	Soft Tissue	Dorsal Midline
Normal [24]	10 kg/cm^2^	12 kg/cm^2^	13 kg/cm^2^
Normal [27]	6.7 kg/cm^2^	7.6 kg/cm^2^	6.1 kg/cm^2^
Back Pain [41]	9 kg/cm^2^	7 kg/cm^2^	na

**Table 9:** Mean MNTs (kg/cm^2^) within spinal regions measured with 1 cm rubber tipped probe.

Condition	Cervical	Thoracic	Lumbar	Pelvic
Normal [24] ^1^	9	12	13	16
Normal [25] ^2^	4.4 ± 0.2	9.0 ± 0.5	6.2 ± 0.4	9.6 ± 0.3
Normal [25] ^2^	4.6 ± 0.1	6.1 ± 0.2	4.7 ± 0.1	7.2 ± 0.3
Normal [43]	na	na	8	10
Ridden [41]	na	8.1 ± 1.0	8.3 ± 0.7	na
Undiagnosed back pain [27] ^1^	5	7	8	11
Back pain [42]	na	4.8 ± 0.2	6.1 ± 0.1	na

^1^ Median values. ^2^ Reported values for two different examiners.

**Table 10:** Mean MNTs (kg/cm^2^) measured at thoracic limb sites.

Site	Lateral	Dorsal	Medial	Palmar	Probe Diameter
Scapula [30]	11.4 ± 0.9	na	na	na	1 cm flat rubber
Shoulder [30]	10.2 ± 1.2	na	na	na	1 cm flat rubber
Elbow [30]	13.4 ± 1.5	na	na	na	1 cm flat rubber
Carpus [30]	15.0 ± 3.1	20.2 ± 3.7	na	na	1 cm flat rubber
Metacarpus [30]	17.2 ± 4.2	24.4 ± 3.9	na	na	1 cm flat rubber
Metacarpus [71]	na	3.1 ± 1.2	na	na	6.5 mm flat metal
Fetlock [30]	17.1 ± 4.2	24.3 ± 4.7	na	na	1 cm flat rubber
First phalanx [71]	na	3.4 ± 1.2	na	na	6.5 mm flat metal
Pastern [30]	27.6 ± 2.3	27.9 ± 2.6	27.5 ± 1.8	19.5 ± 3.6	1 cm flat rubber

**Table 11:** Mean MNTs at different regions within the digit.

Site	Lateral	Dorsal	Medial	Units	Probe Diameter
Coronary band [30]	na	21.2 ± 3.8	na	kg/cm^2^	1 cm flat rubber
Coronary band [71]	na	3.1 ± 1.1	na	kg/cm^2^	6.5 mm flat metal
Coronary band [36]	1.5 ± 0.8	1.5 ± 0.8	1.7 ± 0.1	kg	7 mm flat metal
Heel bulbs [36]	1.7 ± 1.1	na	1.6 ± 0.9	kg	7 mm flat metal
Laminae [36]	na	1.5 ± 1.4 ^1^ 0.9 ± 0.5 ^2^	na	kg	Conical metal
Sole [36]	1.1 ± 0.5	1.2 ± 0.5	1.2 ± 0.6	kg	Conical metal

^1^ Measured in the proximal one-third of the hoof wall. ^2^ Measured at mid-hoof wall.

**Table 12:** Mean MNTs (in Newtons) measured over the heel bulbs in limbs with and without analgesia of the digital flexor tendon sheath [35].

Condition	Normal	Desensitized
Baseline	28 ± 18 (CV = 0.64)	na
Partial desensitization	29 ± 16 (CV = 0.55)	83 ± 25 (CV = 0.30)
Total desensitization	23 ± 18 (CV = 0.78)	130 ± 23 (CV = 0.18)

**Table 13:** Mean MNTs (kgs) for perineural analgesia of the palmar digital nerves [36].

Condition	Dorsal Coronary Band	Lateral Heel Bulb	Medial Heel Bulb	Dorsal Sole	Dorsal Laminae
Baseline	1.50 ± 0.78 (CV = 0.52)	1.71 ± 1.10 (CV = 0.64)	1.60 ± 0.92 (CV = 0.57)	1.23 ± 0.46 (CV = 0.37)	0.90 ± 0.49 (CV = 0.54)
Desensitization ^1^	1.57 ± 0.06 (CV = 0.04)	5.63 ± 0.47 (CV = 0.08)	5.90 ± 0.17 (CV = 0.03)	6 (CV-NA)	5.73 ± 0.25 (CV = 0.04)

^1^ MNT values approximated by the author. Many of these values exceeded the cutoff value of 6 kg.

**Table 14:** Pain sites and conditions with reported MNTs.

Source of Pain
**Acute soft tissue**
Intramuscular neck injections [28]
Microchip insertion [20]
Ventral midline celiotomy [31]
**Acute orthopedic**
Tarsal synovitis [32]
Carpal osteoarthritis [30]
Induced back pain [29]
Naturally occurring acute back pain [38]
**Chronic orthopedic**
Naturally occurring chronic back pain [41,42]
Sacroiliac region pain [18]

**Table 15:** MNT values (kPa) in response to intramuscular injections [28].

Intramuscular Injection	Baseline	Post First Injection	After Last Injection	Treatment Period
Sodium benzylpenicillin	663 ± 202 (CV = 0.30)	415 ± 83 (CV = 0.20)	528 ± 147 (CV = 0.28)	593 ± 350 (CV = 0.59)
Procaine benzylpenicillin	654 ± 197 (CV = 0.30)	603 ± 129 (CV = 0.21)	698 ± 106 (CV = 0.15)	675 ± 208 (CV = 0.31)

**Table 16:** MNT values (kg) in response to microchip insertion [20].

Control	Sham	Microchip
8.56 ± 2.54 (CV = 0.30)	8.88 ± 2.16 (CV = 0.24)	7.95 ± 2.99 (CV = 0.38)

**Table 17:** MNT values (kg/cm^2^) in response to Steinman pin placement [29].

Condition	Non-Instrumented Sites ^1^				Instrumented Sites			
	Pre_1_	Post_1_	Pre_2_	Post_2_	Pre_1_	Post_1_	Pre_2_	Post_2_
Baseline	7.5	10.9	10.0	10.3	7.7	11.9	9.5	9.7
After pin removal	7.7	10.2	9.4	10.5	6.2	8.2	5.8	5.5
Percent change	−3%	6%	6%	−2%	20%	31%	39%	43%

^1^ Pre_1_ and Post_1_ refer to the initial pin placement into T17-T18 and L3-L4 spinous processes. Pre_2_ and Post_2_ refer to a second session of pin placement into the same sites.

**Table 18:** MNT values (% change from baseline) measured 2 weeks post carpal chip induction [30].

Limb	Cervical Region	Proximal Limb	Carpus	Distal Limb	Overall
Control	11	22	12	23	10
Carpal chip	8	20	−7	4	6

**Table 19:** Use of pressure algometry to assess various treatment responses.

Treatment Modality
Chiropractic treatment [37,38,39]
Massage therapy [39] Phenylbutazone administration [39]
Low-level laser therapy [38]
Tail traction [21]
Static magnetic therapy [40]
Pulsed electromagnetic therapy [41]
Extracorporeal shockwave therapy [42]
Ice immersion [34]

## References

[1] Hammarberg M., Egenvall A., Pfau T., Rhodin M. (2016). Rater agreement of visual lameness assessment in horses during lungeing. Equine Vet. J..

[2] Keegan K.G., Dent E.V., Wilson D.A., Janicek J., Kramer J., Lacarrubba A., Walsh D.M., Cassells M.W., Esther T.M., Schiltz P. (2010). Repeatability of subjective evaluation of lameness in horses. Equine Vet. J..

[3] Dyson S., Berger J., Ellis A.D., Mullard J. (2018). Development of an ethogram for a pain scoring system in ridden horses and its application to determine the presence of musculoskeletal pain. J. Vet. Behav..

[4] Luna S.P., Lopes C., Rosa A.C., Oliveira F.A., Crosignani N., Taylor P.M., Pantoja J.C. (2015). Validation of mechanical, electrical and thermal nociceptive stimulation methods in horses. Equine Vet. J..

[5] Vanderweeën L., Oostendorp R.A., Vaes P., Duquet W. (1996). Pressure algometry in manual therapy. Man. Ther..

[6] Keating L., Lubke C., Powell V., Young T., Souvlis T., Jull G. (2001). Mid-thoracic tenderness: A comparison of pressure pain threshold between spinal regions, in asymptomatic subjects. Man. Ther..

[7] Palmer J.L., Bertone A.L. (1994). Experimentally-induced synovitis as a model for acute synovitis in the horse. Equine Vet. J..

[8] Kamerling S.G., Dequick D.J., Weckman T.J., Sprinkle F.P., Tobin T. (1984). Differential effects of phenylbutazone and local anesthetics on nociception in the equine. Eur. J. Pharm..

[9] Brunson D.B., Collier M.A., Scott E.A., Majors L.J. (1987). Dental dolorimetry for the evaluation of an analgesic agent in the horse. Am. J. Vet. Res..

[10] Jöchle W., Hamm D. (1986). Sedation and analgesia with Domosedan (detomidine hydrochloride) in horses: Dose response studies on efficacy and its duration. Acta. Vet. Scand. Suppl..

[11] Szabuniewics M., Szabuniewics J.M. (1975). Use of the hoof tester in diagnosing lameness in horses. Vet. Med. Small Anim. Clin..

[12] Skarda R.T., Muir W.W. (2003). Comparison of electroacupuncture and butorphanol on respiratory and cardiovascular effects and rectal pain threshold after controlled rectal distention in mares. Am. J. Vet. Res..

[13] Mackenzie S.A., Thiboutot E. (1997). Stimulus reactivity tests for the domestic horse (*Equus caballus*). Equine Pr..

[14] Fischer A.A. (1987). Pressure algometry over normal muscles: Standard values, validity and reproducibility of pressure threshold. Pain.

[15] Taylor P.M., Crosignani N., Lopes C., Rosa A.C., Luna S.P., Puoli Filho J.N. (2016). Mechanical nociceptive thresholds using four probe configurations in horses. Vet. Anaesth. Analg..

[16] Hill R.Z., Bautista D.M. (2020). Getting in touch with mechanical pain mechanisms. Trends Neurosci..

[17] Basbaum A.I., Bautista D.M., Scherrer G., Julius D. (2009). Cellular and molecular mechanisms of pain. Cell.

[18] Varcoe-Cocks K., Sagar K.N., Jeffcott L.B., McGowan C.M. (2006). Pressure algometry to quantify muscle pain in racehorses with suspected sacroiliac dysfunction. Equine Vet. J..

[19] Haussler K.K., Behre T.H., Hill A.E. (2008). Mechanical nociceptive thresholds within the pastern region of Tennessee Walking Horses. Equine Vet. J..

[20] Gerber M.I., Swinker A.M., Staniar W.B., Werner J.R., Jedrzejewski E.A., Marcrina A.L. (2012). Health factors associated with microchip insertion in horses. J. Equine Vet. Sci..

[21] Long K., McGowan C.M., Hyytiäinen H.K. (2020). Effect of caudal traction on mechanical nociceptive thresholds of epaxial and pelvic musculature on a group of horses with signs of back pain. J. Equine Vet. Sci..

[22] Malacarne B.D., Cota L.O., Neto A.C.P., Paz C.F.R., Dias L.A., Correa M.G., Carvalho A.M., Faleiros R.R., Xavier A.B.S. (2020). Mechanical nociceptive assessment of the equine hoof following distal interphalangeal joint intra-articular anesthesia. PeerJ.

[23] Pongratz U., Licka T. (2017). Algometry to measure pain threshold in the horse’s back-An in vivo and in vitro study. BMC Vet. Res..

[24] Haussler K.K., Erb H.N. (2006). Mechanical nociceptive thresholds in the axial skeleton of horses. Equine Vet. J..

[25] Menke E.S., Blom G., van Loon J.P.A.M., Back W. (2016). Pressure algometry in Icelandic Horses: Interexaminer and intraexaminer reliability. J. Equine Vet. Sci..

[26] Merrifield-Jones M., Tabor G., Williams J. (2019). Inter- and intra-rater reliability of soft tissue palpation scoring in the equine thoracic epaxial region. J. Equine Vet. Sci..

[27] De Heus P., Van Oossanen G., Van D’ierendonck M.C., Back W. (2010). A pressure algometer is a useful tool to objectively monitor the effect of diagnostic palpation by a physiotherapist in Warmblood horses. J. Equine Vet. Sci..

[28] Olsen L., Bremer H., Olofsson K., Brojer J., Bondesson U., Bergh A., Nostell K., Brostrom H., Bengtsson B., Ingvast-Larsson C. (2013). Intramuscular administration of sodium benzylpenicillin in horses as an alternative to procaine benzylpenicillin. Res. Vet. Sci..

[29] Haussler K.K., Erb H.N. (2006). Pressure algometry for the detection of induced back pain in horses: A preliminary study. Equine Vet. J..

[30] Haussler K.K., Hill A.E., Frisbie D.D., McIlwraith C.W. (2007). Determination and use of mechanical nociceptive thresholds of the thoracic limb to assess pain associated with induced osteoarthritis of the middle carpal joint in horses. Am. J. Vet. Res..

[31] Visser E.M., Menke E.S., van Loon J.P. (2019). Pressure algometry for assessment of abdominal wall sensitivity in horses after ventral midline coeliotomy. Vet. Anaesth. Analg..

[32] Van Loon J.P., Menke E.S., L’Ami J J., Jonckheer-Sheehy V.S., Back W., Rene van Weeren P. (2012). Analgesic and anti-hyperalgesic effects of epidural morphine in an equine LPS-induced acute synovitis model. Vet. J..

[33] Van Loon J.P., Menke E.S., Doornenbal A., Back W., Hellebrekers L.J. (2012). Antinociceptive effects of low dose lumbosacral epidural ropivacaine in healthy ponies. Vet. J..

[34] Schambourg M., Taylor P.M. (2020). Mechanical nociceptive thresholds in endurance horses. Vet. Rec..

[35] Jordana M., Martens A., Duchateau L., Vanderperren K., Saunders J., Oosterlinck M., Pille F. (2014). Distal limb desensitisation following analgesia of the digital flexor tendon sheath in horses using four different techniques. Equine Vet. J..

[36] Paz C.F.R., Magalhães J.F., Mendes H.M.F., Rocha Junior S., Belknap J.K., Alves G.E.S., Faleiros R.R. (2016). Mechanical nociceptive thresholds of dorsal laminae in horses after local anaesthesia of the palmar digital nerves or dorsal branches of the digital nerve. Vet. J..

[37] Haussler K.K., Erb H.N. (2003). Pressure algometry: Objective assessment of back pain and effects of chiropractic treatment. Proc. 49th Annu. Conv. Am. Assoc. Equine Pract..

[38] Haussler K.K., Manchon P.T., Donnell J.R., Frisbie D.D. (2020). Effects of low-level laser therapy and chiropractic care on back pain in Quarter Horses. J. Equine Vet. Sci..

[39] Sullivan K.A., Hill A.E., Haussler K.K. (2008). The effects of chiropractic, massage and phenylbutazone on spinal mechanical nociceptive thresholds in horses without clinical signs. Equine Vet. J..

[40] Edner A., Lindberg L.G., Brostrom H., Bergh A. (2015). Does a magnetic blanket induce changes in muscular blood flow, skin temperature and muscular tension in horses?. Equine Vet. J..

[41] Biermann N.M., Rindler N., Buchner H.H.F. (2014). The effect of pulsed electromagnetic fields on back pain in polo ponies evaluated by pressure algometry and flexion testing: A randomized, double-blind, placebo-controlled trial. J. Equine Vet. Sci..

[42] Trager L.R., Funk R.A., Clapp K.S., Dahlgren L.A., Werre S.R., Hodgson D.R., Pleasant R.S. (2020). Extracorporeal shockwave therapy raises mechanical nociceptive threshold in horses with thoracolumbar pain. Equine Vet. J..

[43] L’Ami J.J., Vermunt L.E., van Loon J.P., Sloet van Oldruitenborgh-Oosterbaan M.M. (2013). Sublingual administration of detomidine in horses: Sedative effect, analgesia and detection time. Vet. J..

[44] Starling M., McLean A., McGreevy P. (2016). The Contribution of Equitation Science to Minimising Horse-Related Risks to Humans. Animals.

[45] Love E.J., Taylor P.M., Murrell J., Whay H.R. (2012). Effects of acepromazine, butorphanol and buprenorphine on thermal and mechanical nociceptive thresholds in horses. Equine Vet. J..

[46] Chambers J.P., Waterman A.E., Livingston A. (1994). Further development of equipment to measure nociceptive thresholds in large animals. J. Vet. Anaesth..

[47] Nalon E., Maes D., Piepers S., van Riet M.M.J., Janssens G.P.J., Millet S., Tuyttens F.A.M. (2013). Mechanical nociception thresholds in lame sows: Evidence of hyperalgesia as measured by two different methods. Vet. J..

[48] Duan G., Xiang G., Zhang X., Guo S., Zhang Y. (2014). An improvement of mechanical pain sensitivity measurement method: The smaller sized probes may detect heterogeneous sensory threshold in healthy male subjects. Pain Med..

[49] Grint N.J., Beths T., Yvorchuk K., Taylor P.M., Dixon M., Whay H.R., Murrell J.C. (2014). The influence of various confounding factors on mechanical nociceptive thresholds in the donkey. Vet. Anaesth. Analg..

[50] Polson S., Taylor P.M., Yates D. (2014). Effects of age and reproductive status on postoperative pain after routine ovariohysterectomy in cats. J. Feline. Med. Surg..

[51] Grint N.J., Beths T., Yvorchuk-St Jean K., Whay H.R., Murrell J.C. (2017). Analysis of behaviors observed during mechanical nociceptive threshold testing in donkeys and horses. J. Equine Vet. Sci..

[52] Koo T.K., Guo J.Y., Brown C.M. (2013). Test-retest reliability, repeatability, and sensitivity of an automated deformation-controlled indentation on pressure pain threshold measurement. J. Manip. Physiol..

[53] Linde L.D., Kumbhare D.A., Joshi M., Srbely J.Z. (2018). The relationship between rate of algometer application and pain pressure threshold in the assessment of myofascial trigger point sensitivity. Pain Pr..

[54] Kinser A.M., Sands W.A., Stone M.H. (2009). Reliability and validity of a pressure algometer. J. Strength Cond. Res..

[55] Vaughan B., McLaughlin P., Gosling C. (2007). Validity of an electronic pressure algometer. Int. J. Osteopath. Med..

[56] Spadavecchia C., Arendt-Nielsen L., Andersen O.K., Spadavecchia L., Doherr M., Schatzmann U. (2003). Comparison of nociceptive withdrawal reflexes and recruitment curves between the forelimbs and hind limbs in conscious horses. Am. J. Vet. Res..

[57] Colbath A.C., Wittenburg L.A., Gold J.R., McIlwraith C.W., Moorman V.J. (2016). The effects of mepivacaine hydrochloride on antimicrobial activity and mechanical nociceptive threshold during amikacin sulfate regional limb perfusion in the horse. Vet. Surg..

[58] Lizarraga I., Beths T. (2012). A comparative study of xylazine-induced mechanical hypoalgesia in donkeys and horses. Vet. Anaesth. Analg..

[59] Hinnigan G., Milner P., Talbot A., Singer E. (2014). Is anaesthesia of the deep branch of the lateral plantar nerve specific for the diagnosis of proximal metatarsal pain in the horse?. Vet. Comp. Orthop. Traumatol..

[60] List T., Helkimo M., Karlsson R. (1991). Influence of pressure rates on the reliability of a pressure threshold meter. J. Craniomandib. Disord..

[61] Treede R.D., Rolke R., Andrews K., Magerl W. (2002). Pain elicited by blunt pressure: Neurobiological basis and clinical relevance. Pain.

[62] Gozalo-Marcilla M., de Oliveira A.R., Fonseca M.W., Possebon F.S., Pelligand L., Taylor P.M., Luna S.P.L. (2019). Sedative and antinociceptive effects of different detomidine constant rate infusions, with or without methadone in standing horses. Equine Vet. J..

[63] Love E.J., Pelligand L., Taylor P.M., Murrell J.C., Sear J.W. (2015). Pharmacokinetic-pharmacodynamic modelling of intravenous buprenorphine in conscious horses. Vet. Anaesth. Analg..

[64] Nussbaum E.L., Downes L. (1998). Reliability of clinical pressure-pain algometric measurements obtained on consecutive days. Phys. Ther..

[65] Kosek E., Ekholm J., Nordemar R. (1993). A comparison of pressure pain thresholds in different tissues and body regions. Long-term reliability of pressure algometry in healthy volunteers. Scand. J. Rehabil. Med..

[66] Pauli P., Wiedemann G., Nickola M. (1999). Pressure pain thresholds asymmetry in left- and right-handers: Associations with behavioural measures of cerebral laterality. Eur. J. Pain.

[67] Rédua M.A., Valadão C.A.A., Duque J.C., Balestrero L.T. (2002). The pre-emptive effect of epidural ketamine on wound sensitivity in horses tested by using von Frey filaments. Vet. Anaesth. Analg..

[68] Mothershead M.L. The Effect of Weight Carried and Time Ridden on Back Pain in Horses Ridden During Horse Shows as Determined by Pressure Algometry. https://bearworks.missouristate.edu/theses/3206/.

[69] Hamra J.G., Kamerling S.G., Wolfsheimer K.J., Bagwell C.A. (1993). Diurnal variation in plasma ir-beta-endorphin levels and experimental pain thresholds in the horse. Life Sci..

[70] Ohrbach R., Gale E.N. (1989). Pressure pain thresholds in normal muscles: Reliability, measurement effects, and topographic differences. Pain.

[71] Crabtree N.E., Mochal-King C.A., Sloan P.B., Eddy A.L., Wills R.W., Meredith A.N., Fontenot R.L. (2019). Synovial butorphanol concentrations and mechanical nociceptive thresholds after intravenous regional limb perfusion in standing sedated horses. Vet. Surg..

[72] Kosek E., Ekholm J., Hansson P. (1999). Pressure pain thresholds in different tissues in one body region. The influence of skin sensitivity in pressure algometry. Scand. J. Rehabil. Med..

[73] Jordana M., Martens A., Duchateau L., Haspeslagh M., Vanderperren K., Oosterlinck M., Pille F. (2016). Diffusion of mepivacaine to adjacent synovial structures after intrasynovial analgesia of the digital flexor tendon sheath. Equine Vet. J..

[74] Contino E.K., King M.R., Valdes-Martinez A., McIlwraith C.W. (2015). In vivo diffusion characteristics following perineural injection of the deep branch of the lateral plantar nerve with mepivacaine or iohexol in horses. Equine Vet. J..

[75] Muir W.W. (2010). Pain: Mechanisms and management in horses. Vet. Clin. Equine Pr..

